# A preventable problem in pregnancy

**DOI:** 10.1007/s40620-025-02274-w

**Published:** 2025-04-21

**Authors:** Kareem Elbosaty, Alaa Sabry, Giorgina B. Piccoli, Rasha Shemies

**Affiliations:** 1 Mansoura Nephrology and Dialysis Unit, Mansoura University, Mansoura, Egypt; 2 Centre Hospitalier Le Mans, Le Mans, France

**Keywords:** Heparin, Renal tubular acidosis, Pregnancy, Skin necrosis & thrombocytopenia

The image in Fig. 1a shows extensive heparin-induced skin necrosis. The photograph was taken when a 30-year-old woman was referred to Mansoura University Hospital in Egypt at the 37th gestational week of her 5th pregnancy, with high fever (Temp 38.7 °C), low blood pressure (85/50 mmHg), respiratory rate 26/min, and heart rate 103/min. She had a history of renal tubular acidosis, diagnosed 7 years previously (treated with oral potassium and bicarbonate supplements, with signs of nephrocalcinosis at CT scan; adherence had been irregular in the past, but was optimal during pregnancy), well-controlled hypothyroidism, diagnosed one year previously (on Levothyroxine 50 µg/day). At referral she had severe hypokalaemia (potassium 1.5 mmol/L, bicarbonate 12.2 mmol/L, CRP 48 mg/dl, leukocyte count 10.9 × 109/L, platelets 219 × 109/L). Caesarean section under spinal anaesthesia was performed after hemodynamic stabilisation, antibiotic treatment and potassium replacement; a viable, healthy, full-term baby was then delivered. The patient had married at the age of 18, and had one child at the age of 23, and three first-trimester miscarriages, one at the age of 19 and two at the age of 25.

**Fig. 1:**
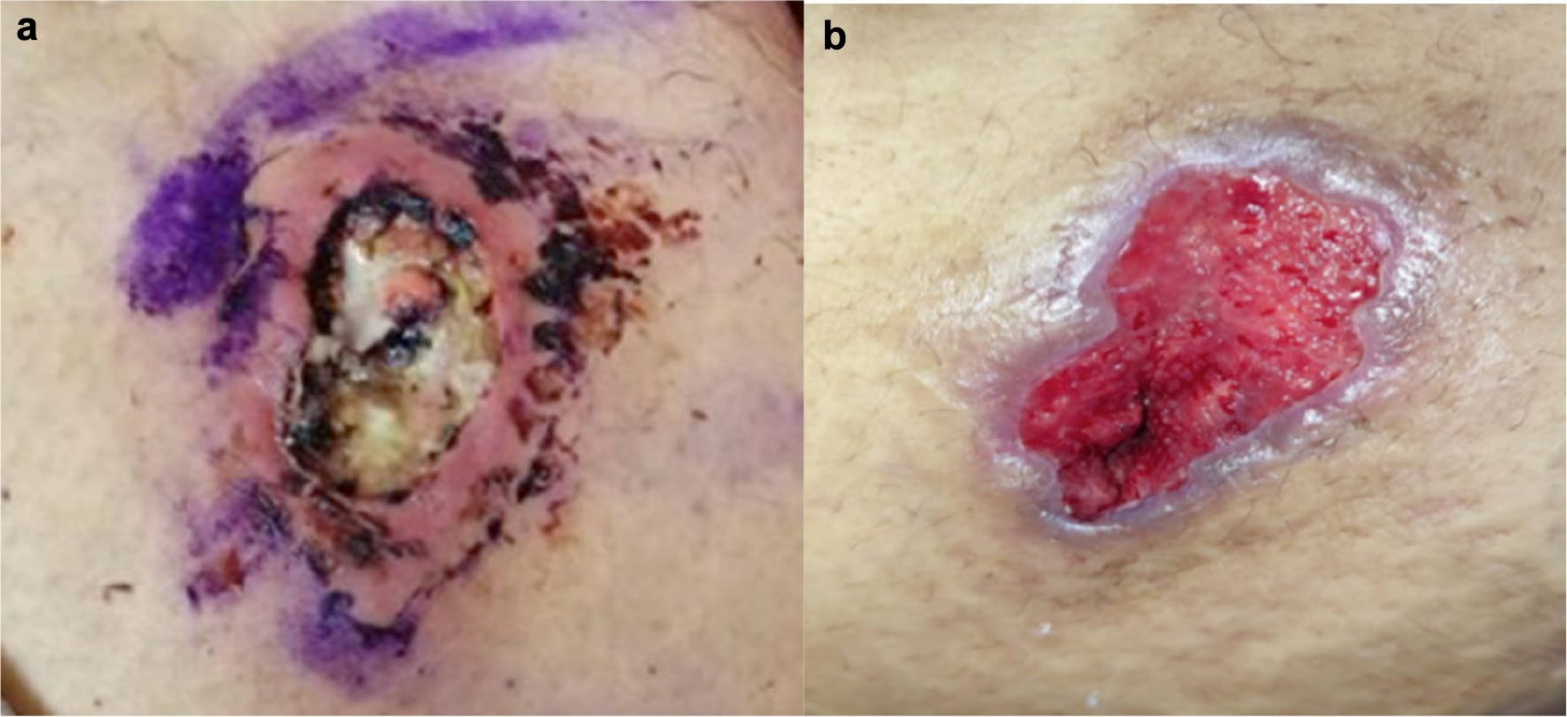
**a**: extensive heparin-induced abdominal necrosis with some adjacent at-risk areas. The purple marks indicate staining by the gentian violet dye the patient purchased over-the-counter to use as an antiseptic (size 5 × 3 cm). **b**: recovering lesion after discontinuation of heparin and debridement

The lesion in Fig. 1 developed over the four weeks prior to her admission to hospital. Late referral was motivated by the fear of discontinuing a “miracle medication”, 5000 units of unfractionated heparin every other day, that was prescribed at the start of pregnancy and was thought to prevent miscarriages. The prescription of heparin during pregnancy is indicated in patients with, or at risk of, thromboembolic disease. Non-fractionated heparin, although less expensive, may lead to complications such as haemorrhage, heparin-induced thrombocytopaenia and osteoporosis [1]. Heparin-induced cutaneous necrosis is a rare, potentially severe adverse event whose pathogenesis is not fully explained by local injection-mediated trauma. While often associated with heparin-induced thrombocytopenia, the lesions, typically in the setting of repeated injections, may occur in the presence of normal platelet counts, and are thought to be due to delayed-type hypersensitivity [2, 3]. The diagnosis is usually clinical, due to the risk of both extending the necrosis if adjacent tissue is biopsied, and low diagnostic yield of biopsy in necrotic areas. Discontinuation of heparin, antibiotic therapy and debridement, together with potassium and bicarbonate supplementation, led to rapid clinical improvement (Fig. 1b).

In settings like Egypt, where successful pregnancy is culturally important, erring on the side of caution may lead to unreasonable overtreatment, as it did in this case, in which unfractionated heparin was prescribed without any sound indication, and, in any case, at ineffective doses.

Education aimed at avoiding ineffectual and potentially dangerous treatments, with the aim of “protecting” a pregnancy is crucially needed to protect women from adverse events during pregnancy.

## Data Availability

Data are available upon request from the journal.
